# An Investigation of the Ground Walnut Shells’ Addition Effect on the Properties of the Fly Ash-Based Geopolymer

**DOI:** 10.3390/ma15113936

**Published:** 2022-05-31

**Authors:** Barbara Kozub, João Castro-Gomes

**Affiliations:** 1 Department of Materials Engineering, Faculty of Materials Engineering and Physics, Cracow University of Technology, Al. Jana Pawła II 37, 31-864 Cracow, Poland; 2 C-MADE Centre of Materials and Building Technologies, University of Beira Interior, 6201-001 Covilhã, Portugal; castro.gomes@ubi.pt

**Keywords:** fly ash, geopolymer, ground walnut shells, efflorescence, thermal conductivity, water absorption

## Abstract

The development of geopolymers is in line with the requirements of sustainable development. Creating a new type of material from various industrial and bio-based wastes and by-products can lead to reduced energy consumption, reduced waste generation, reduced global CO_2_ emissions, as well as reduced resource extraction of natural resources. In this study, geopolymer composites based on class F fly ash with the addition of fine quartz sand and ground walnut shells used as a substitute for sand were examined. The study focused on investigating the effects of different weight percentages of ground walnut shells and quartz sand on the density and strength properties, including compressive and flexural strength, thermal conductivity, efflorescence formation, and water absorption of the fly ash-based geopolymer composites. The microstructure of the studied geopolymers was also analyzed using a scanning electron microscope (SEM). It was observed that the addition of ground walnut shells contributes to the decrease in density and mechanical properties, increase in absorption properties, and decrease in porosity of fly ash-based geopolymers. Furthermore, the addition of ground walnut shells allows for a significant reduction in efflorescence on the surface of the tested geopolymer composites. Moreover, partial or complete replacement of sand by ground walnut shells in geopolymer composites based on fly ash allows for a significant reduction in their thermal conductivity, which makes it possible to use these composites as insulation materials.

## 1. Introduction

In recent years, geopolymer materials have aroused more and more interest both in the scientific community and in various areas of industry, where they can be used [1,2,3]. Geopolymers belong to the group of synthetic inorganic polymers, which are obtained in the process of alkaline activation of aluminosilicates, e.g., fly ash, metakaolin, red mud, and others [4,5,6]. 

Fly ash is one of the raw materials most often used in the production of geopolymers. According to the EN-450-1: 2012 [7] standard, fly ash is defined as fine-grained dust resulting from the combustion of coal dust, which mainly consists of vitrified, spheroidal particles. The fly ash consists mainly of silicon dioxide (SiO_2_) and aluminum oxide (Al_2_O_3_). Fly ash is characterized by puculanic properties [7]. Fly ash obtained in Poland is obtained from the combustion process carried out in conventional CHP (Combined Heat and Power) furnaces at a temperature of 1200–1400 °C of coal dust [8,9,10].

Because of the mechanical properties, as well as corrosion resistance and high thermal resistance, geopolymers are increasingly used in various industries and are considered a potential substitute for Portland cement. In addition, a vast source of raw materials, lower energy requirements, and lower CO_2_ emissions in comparison with conventional concretes are of great importance here. The properties of the geopolymer depend on the type of the base material and the type and amount of activator used in its production. Additionally, external factors (temperature and heating time) of the polycondensation process can be controlled, which also affects the subsequent properties of the resulting material [11].

Compared with composites based on conventional cement, geopolymer composites are characterized by higher durability, corrosion resistance, and higher resistance at elevated temperatures [12]. However, because of the brittleness of geopolymers, they have relatively low flexural strength and tensile stress and show high susceptibility to microcracks. These problems can be eliminated, or the selected properties of geopolymers can be improved by adding to them fibers, which, above all, can significantly reduce the propagation of microcracks while improving their ductility, toughness, and tensile strength. In the absence of fiber reinforcements, cracks under load can propagate rapidly and lead to loss of load-bearing capacity, whereas the use of fibers as reinforcements can cause the fibers to stop the crack, slowing or even preventing further expansion. This is known as the crack masking effect, in which the concrete hardness increases and the material retains its load-bearing capacity even after the first crack has formed [12,13,14].

Fiber-reinforced geopolymers have better durability compared with conventional cement of the same grade [15]. Therefore, in recent years, many scientists have conducted numerous studies focusing on the study of the effect of the addition of various types of fibers on the properties of geopolymer composites [16,17]. One of the most commonly used are natural fibers (including cotton [18,19], banana [20], sisal [21], basalt [22,23], etc.), organic (including polypropylene [24], polyethylene [25], etc.) and inorganic (including steel fibers [26], glass fibers [27], carbon fibers [28], etc.).

In recent years, more attention has been paid to aggregates of natural origin, which can be used in the production of lightweight geopolymer composites. The use of such aggregates is considered environmentally friendly since these aggregates are recyclable [29,30,31]; therefore, they are used to replace synthetic fibers. Replacing artificial fibers with natural equivalents can significantly reduce the so-called carbon footprint of the product, especially the reduction in CO_2_ emissions. However, the reduced environmental impact is not the only advantage of natural fibers. These fibers also have other properties that make them easy to use as reinforcements in a variety of composite materials, including plastics and concrete. The most important advantages include repeatability of raw materials, including in many cases relatively short “production” times (short plant vegetation cycles) and relatively low production costs. Compared with chemical fibers, they have low density and high specific strength, they are nontoxic to the human body and environmentally friendly, and are usually easy to process [19,30,31,32,33]. Unfortunately, in the case of natural aggregates, obstacles are still encountered in the geopolymer production process, the most difficult of which is the decomposition of some chemical components of the aggregate, which occurs in an alkaline environment, which in turn results in the weakening of the bond between the aggregate and the geopolymer matrix [34,35,36]. Whether this problem can be avoided will depend on the type, form, and properties of the selected wood aggregate. 

The development of geopolymers is in line with the requirements of sustainable development. Not only does it require lower processing temperatures, but it can also use a significant amount of industrial and bio-based waste as secondary raw material and convert it into a new product. Creating a new type of material from various industrial and agricultural wastes and by-products can lead to reduced energy consumption, reduced waste generation, reduced global CO_2_ emissions, as well as reduced resource extraction of natural resources. The agricultural sector generates large amounts of waste and by-products that can be a source of raw materials for various industries, including the production of building materials. Walnut shells are agricultural by-products/waste that are incinerated or disposed of in landfills. According to the report [37], the world production of walnuts in 2019 was about 965,000 tons. As the shells are estimated to account for almost 67% of the fruit’s weight, this is equivalent to 646.818 tons of walnut shells per year. Therefore, because of their profitability and environmental friendliness, they can find real application in the construction industry, at the same time being in line with the assumptions of the sustainable development economy [38].

The main objective of the research is to assess the feasibility of using waste ground walnut shells as a substitute for fine aggregate (quartz sand) in the production of geopolymer mortars. It is anticipated that the use of ground walnut shells will result in the production of lightweight geopolymer composites. To conduct the research, geopolymer composites based on fly ash with the addition of fine quartz sand and ground walnut shells used as a sand substitute were made. The research participants focused on examining the effect of the weight percentage of ground walnut slates on selected properties. The scope of the research carried out included the measurements of density, compressive and bending strength, thermal conductivity measurements, water absorption tests, visual assessment of efflorescence, and microstructure observation with the use of a scanning electron microscope (SEM).

## 2. Materials and Methods

### 2.1. Materials

**Figure 1:**
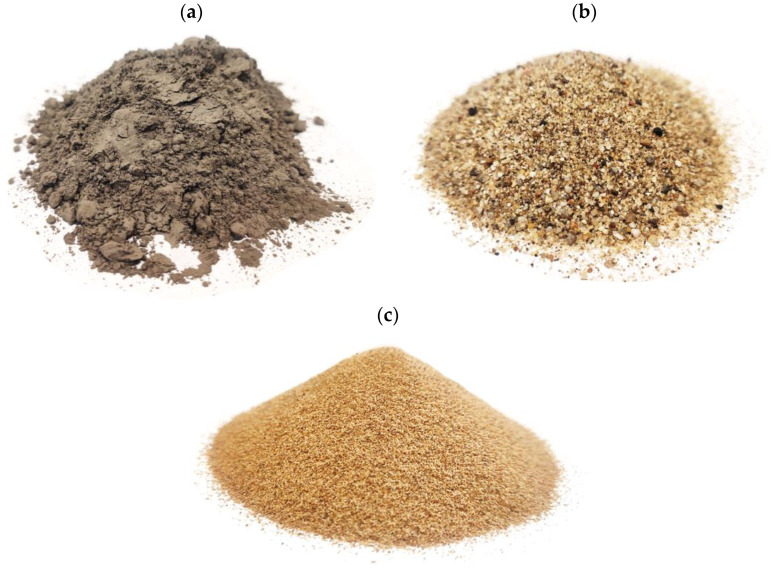
(**a**) Fly ash; (**b**) quartz sand; (**c**) ground walnut shells.

Tests were conducted on geopolymer composites with a matrix based on class F fly ash with the addition of fine quartz sand and ground walnut shells used as a substitute for sand (Figure 1).

**Figure 2:**
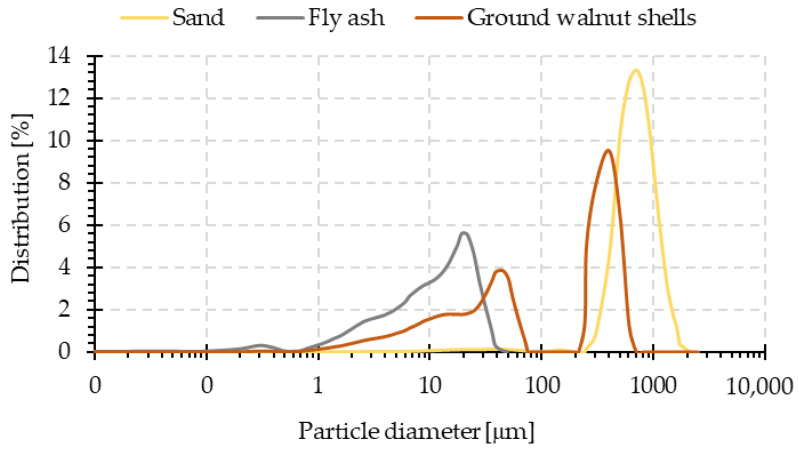
Particle size distribution of fly ash, sand, and ground walnut shells.

**Figure 3:**
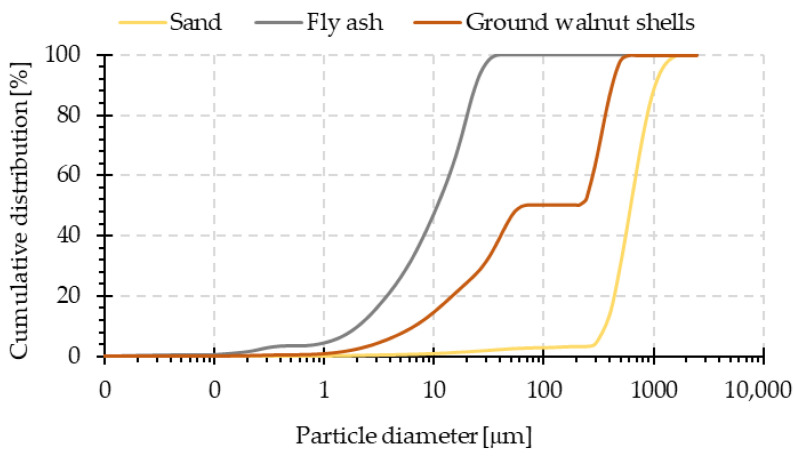
Cumulative curves of the particle size distribution of fly ash, sand, and ground walnut shells.

The fly ash used in this study was obtained from the Skawina Combined Heat and Power Plant (Skawina, Poland). The ash mainly consists of aluminum oxide (Al_2_O_3_) and silicon dioxide (SiO_2_), while the content of calcium oxide (CaO) does not exceed 4%. The exact percentages of the phases constituting the examined ash were presented in the previous paper [39]. This fly ash, because of its composition, especially its high content of aluminum and silicon, as well as physicochemical properties (mostly fine particle fraction–Figure 2—fineness of 16.7% and density 2.22 g/cm^3^), can be successfully used in the geopolymerization process [40,41]. Because of the surface saturation of the particles, the sand used in this study did not exhibit surface absorption. The ground walnut shells used in this study, from Herubin (Dobra, Poland), consist of cellulose (55–70 wt.%), lignin (19–22 wt.%), and hemicellulose (22–27 wt.%). The density of ground walnut shells is about 1.28 g/cm^3^, the water content is 8.7 wt.%, and the Mohs hardness is 2.5–3.0. Figure 2 and Figure 3 show particle size distribution plots and cumulative curves for all solid components used in the study. The presented results come from own research carried out on the particle size analyzer from Anton Paar GmbH (Graz, Austria).

### 2.2. Preparation of Specimens

The solid raw materials, i.e., fly ash, quartz sand, and ground walnut shells, were mixed in different ratios, as shown in Table 1. A reference sample was also made for which fly ash and quartz sand were used in a 1:1 ratio.

The activation process was carried out using a 10 molar solution of sodium hydroxide (NaOH) and an aqueous solution of sodium silicate R-145 (2.5 molar modulus; density about 1.45 g/cm^3^), with a 1:2 ratio of sodium hydroxide to sodium silicate solutions. To prepare the activator, solid sodium hydroxide flakes were dissolved in water (tap water was used for this study), and then sodium silicate solution was added to the prepared solution. The mixed alkaline solution was allowed to equilibrate to its ambient temperature. The solid components (measured in appropriate proportions) together with the prepared solution were mixed for 15 min in a GEOLAB cement mortar mixer (GEOLAB, Warsaw, Poland) until a homogeneous paste was obtained. The final step in sample preparation was to cast the geopolymer paste into molds, which were then placed on a vibrating table to remove air bubbles from the paste. The curing process of the geopolymer composites was carried out at 75 °C for 24 h in a SLW 750 STD laboratory dryer (POL-EKO-APARATURA, Wodzisław Śląski, Poland). The cured samples were removed from the molds and stored under ambient conditions. The samples were examined after 28 days.

### 2.3. Methods

Density measurements were made on a series of six cuboid specimens with dimensions of 50 × 50 × 50 mm for each of the tested compositions before the compressive strength test. The standard deviation was calculated for all the obtained results and plotted as error bars on the graphs. The density was determined using a geometrical method based on dimensions (measurement with laboratory caliper with 0.01 mm accuracy) and sample mass determined on RADWAG PS 200/2000.R2 laboratory balance (maximum load: 200/2000 g; reading accuracy: 0.001/0.01 g) produced by Radwag (Radom, Poland).

**Figure 4:**
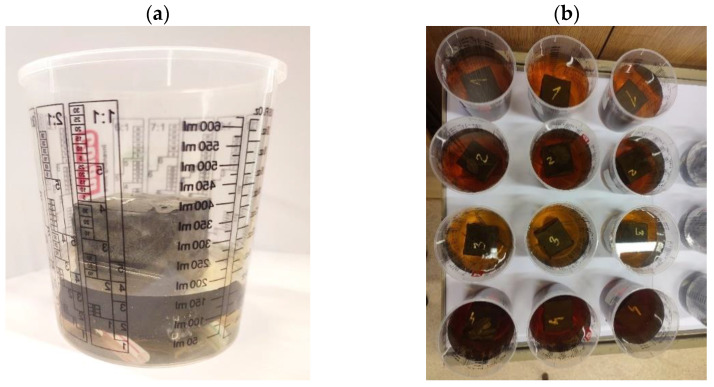
(**a**) Example sample prepared for water absorption testing—1st day of measurements (flooding the sample to about half of its height); (**b**) samples fully immersed in distilled water 24 h after starting the tests.

Perpendicular specimens with dimensions of 50 × 50 × 50 mm were used for water absorption testing. Three specimens were tested for each of the tested geopolymer compositions so that the standard deviation was calculated, shown as error bars on the graph. Distilled water was used for testing. Initially, the specimens were flooded to about half their height, and after 24 h, the specimens were weighed and then flooded with water so that they were completely below the water surface (Figure 4).

Samples were weighed daily for the first seven days, with subsequent measurements taken on days 14 and 28 after the samples were first flooded. For measurements, samples were removed from the water, wiped with filter paper to remove water from their surfaces, and weighed on a laboratory balance. The samples were again submerged in water to continue sorption until saturation. Each time weighing was done in about 30 s to avoid error due to water evaporation. Water content (*M_t_*), expressed as a percentage, was determined according to the following relationship [42]:

(1) $$M_{t}=\left(\frac{W_{t}-W_{0}}{W_{0}}\right)\cdot 100$$

where *W_t_* is the weight of the sample at time *t* (g), and *W*_0_ is the initial weight of the sample (g).

The same types of samples as for the water adsorption tests were placed in containers and poured to about half the height of each sample. The samples prepared in this way were left in the containers with water, and after 28 days, the samples were visually inspected. Observations were made for all tested geopolymer compositions.

Compressive strength tests of the tested geopolymer composites were performed according to EN 12390-3 [43] on a Matest 3000 kN universal testing machine (Matest, Treviolo, Italy). The measurement was performed at a speed of 0.05 MPa/s. The dimensions of the specimens prepared for testing were 50 × 50 × 50 mm. The specimens were tested after 28 days of seasoning in the laboratory under ambient conditions. The measurements were carried out on a series of six samples for each of the tested compositions, both nonsoaked (dry) and samples after water absorption tests. For all of the obtained results, the standard deviation was calculated and plotted on the graphs in the form of error bars. Compressive strength was determined according to the following equation:

(2) $$f_{c}=\frac{F}{A_{c}}$$

where *f_c_* is the compressive strength [MPa], *F* is maximum load [N], and *A_c_* is the cross-sectional area of the sample (mm).

Flexural strength tests of the tested geopolymer composites were performed according to EN 12390-5 [44] also on a Matest 3000 kN testing machine. The measurement was performed at a speed of 0.05 MPa/s. The dimensions of the specimens prepared for testing were 50 × 50 × 200 mm, while the distance between the support points was equal to 150 mm. The measurements were carried out on a series of four specimens for each of the tested compositions. The standard deviation was calculated for all of the obtained results and plotted as error bars on the graphs. The bending strength was determined according to the following equation:

(3) $$f_{f}=\frac{3\cdot F\cdot I}{2\cdot d_{1}\cdot d_{2}^{2}}$$

where: *f_f_* is the bending strength [MPa], *F* is maximum load [N], *I* is the distance between support rollers (mm), and *d*_1_ and *d*_2_ are transverse dimensions of the sample [mm].

Thermal conductivity coefficient measurements were performed under ASTM C518 JIS A1412 [45], ISO 8301 [46], and DIN EN 12667 [47] on an HFM 446 Lambda Series instrument from NETZSCH (Netzsch GmbH & Co., Selb, Germany). For the tests, 200 × 200 × 25 mm specimens were made for each of the tested geopolymer compositions, with the center of the 100 × 100 mm specimen used for analysis. Measurements were made for three temperature ranges: 0–20 °C, 20–40 °C, and 30–50 °C.

Cross-sectional observations of the tested composites were performed using a JEOL JSM-820 scanning electron microscope EDS (IXR Inc., Austin, TX, USA). The microscope observations were performed for both dry samples and samples after water absorption tests. Before imaging, all samples were sputtered with gold.

## 3. Results and Discussion

### 3.1. Efflorescences

**Figure 5:**
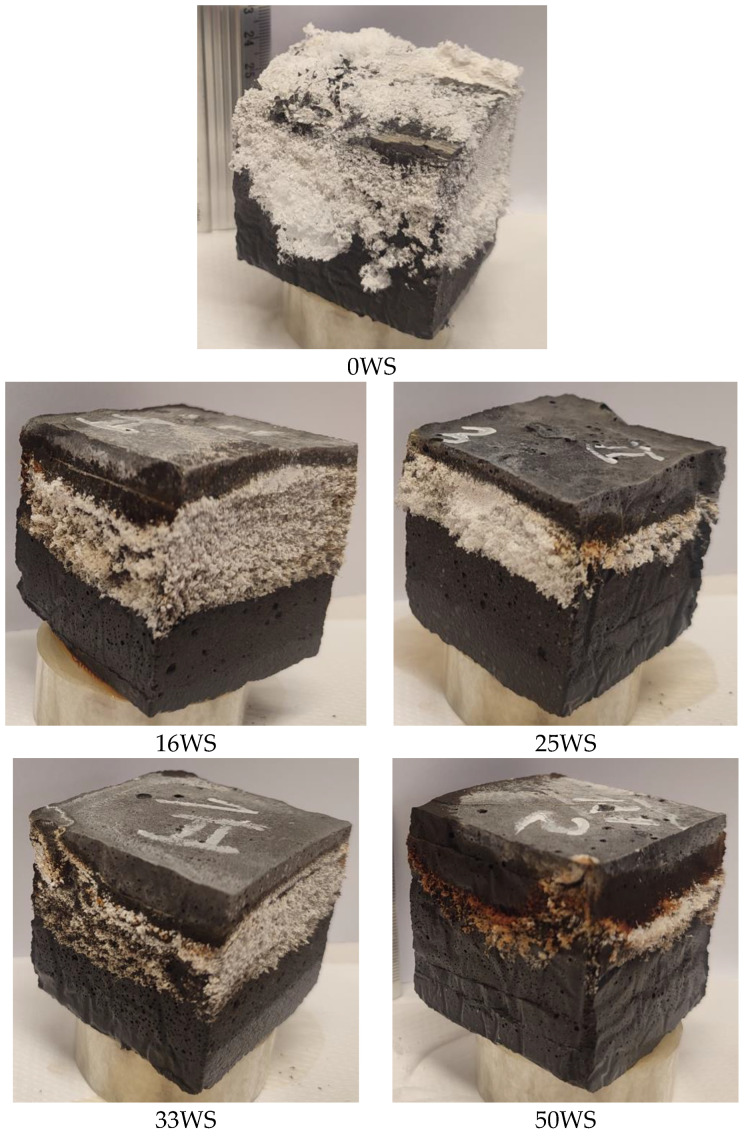
Efflorescence formed on the surface of the tested geopolymer composites.

Figure 5 shows the results of the visual evaluation of efflorescence for the tested geopolymer compositions. The occurrence of efflorescence was observed for all tested samples, with the amount and location of efflorescence largely dependent on the number of ground walnut shells in the composite. In the case of reference samples, in which the composition did not contain ground walnut shells, the greatest development of efflorescence was observed, both on the sidewalls and on the upper surface of the samples. However, for the other compositions with the addition of walnut shells, efflorescence appeared directly above the waterline with a marked reduction on the upper surface. This is most likely related to the “closing” of the capillary pore system by ground walnut shells. A similar effect during water absorption testing of fly ash-based geopolymer composites with coffee grounds was obtained by Mierzwiński et al. [48]. Moreover, the addition of ground walnut shells could cause a diminish in pH, and as has been shown in the literature [48], some additives, such as coffee fusions, eliminate or limit the efflorescence effect by pH reduction. However, further research is required to confirm this hypothesis.

In the case of concrete, efflorescence occurs due to carbonation of calcium hydroxide-Ca(OH)_2_, also known as portlandite, which is transported through the system of capillary pores in the structure of concrete and penetrates its surface layer. The main factors affecting the rate and intensity of carbonation are humidity, and ambient carbon dioxide concentration-carbonation occurs most intensively when the surface of capillaries is covered with a thin film of water, which allows for rapid diffusion of carbon dioxide, which dissolves and reacts with calcium ions [49]. Moreover, high porosity [50,51], low density of the concrete mixture, and the method of concrete care [52,53] are mentioned as additional factors promoting carbonation in the literature. An analogous mechanism is encountered in the case of geopolymers; however, because of their chemical composition, the appearance of sodium carbonate heptahydrate on their surface may occur [54].

In the case of geopolymers, efflorescence occurs due to the reaction of Na with the CO_2_ in the atmosphere to form Na_2_CO_3_. Efflorescence occurs when poorly reactive materials are used as precursors and therefore do not fully combine with the Na present in the activation solution. The more unreacted Na, the more extensive the efflorescence occurrence. Similar efflorescence phenomena occur in the presence of potassium in a system with K as an activator (instead of Na), forming potassium carbonate (K_2_CO_3_) [55]. Moreover, adding reactive alumina-silicate-rich materials in the blended systems prevents efflorescence phenomena by generating more alkaline activation reaction products [56].

However, special attention should be paid to the fact that in the case of conventional concrete, according to PN-EN 1338:2005 [57], efflorescence is allowed, while in the case of geopolymers, the presence of efflorescence may adversely affect the mechanical properties [48,58].

### 3.2. Water Absorption

**Figure 6:**
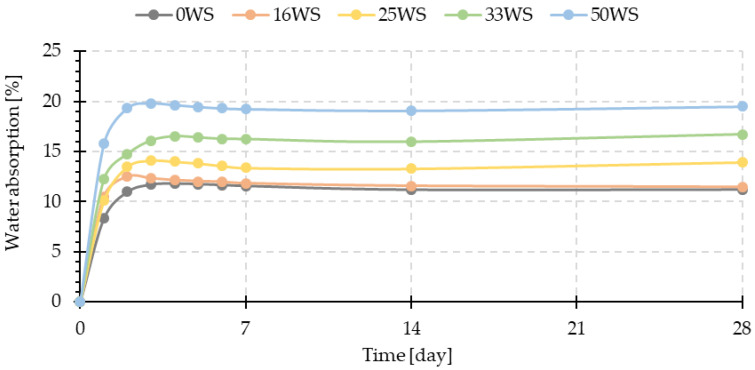
Water absorption of tested geopolymer composites.

Figure 6 shows the curves obtained from the water absorption tests of the tested geopolymer composites. It can be shown that water absorption for all the tested composites occurred most intensively during the first two days of testing. It can be seen that the samples with ground walnut shells exhibit a significantly higher water absorption capacity compared with the fly ash and sand-based geopolymer. The amount of ground walnut shells introduced in place of sand has a significant effect on the water absorbency-the higher the weight proportion in the mixture, the better the absorbency properties of the geopolymer composite showed, but for any of the tested samples, the water absorption does not exceed 20%. Cellulose and hemicellulose are highly hydrophilic materials, while lignin has hydrophobic properties. The ground walnut shells used in the research consist mainly of cellulose and hemicellulose and 19–22 wt.% from lignin; therefore, it can be assumed that they, like wood aggregates [29,30], are mainly characterized by hydrophilic properties, which favors an increase in water soaking in geopolymer composites with their addition. On day 28 of the study, the composite in which the ratio of fly ash to ground walnut shells was 1:1 (50WS-there was no sand in the composition) showed about 74% higher water absorption compared with the reference sample (0WS).

Similarly, the addition of coffee grounds contributes to increasing the absorption properties of fly ash-based geopolymer composites. At the same time, in the case of coffee grounds, their amount in the composite does not have a significant effect on the results obtained; there is more intense evaporation of water because of increased porosity [45].

Sarmin et al. [29] presented in their work the results of water absorption of geopolymer composites based on fly ash and metakaolin with 10% addition of different types of wood aggregates, i.e., wood particles, wood flour, and wood fibers. As shown in the results, the composites with the addition of wood aggregates showed better absorption properties compared with the reference sample, and similar to the results presented in this paper, water absorption occurred most intensively in the early stages of exposure.

Also, Alomayri et al. [30] and Dhakal et al. [59] indicated in their work that the hydrophilic nature of wood aggregates contributes to the absorption properties of geopolymer composites with their addition.

### 3.3. Density and Morphology

**Figure 7:**
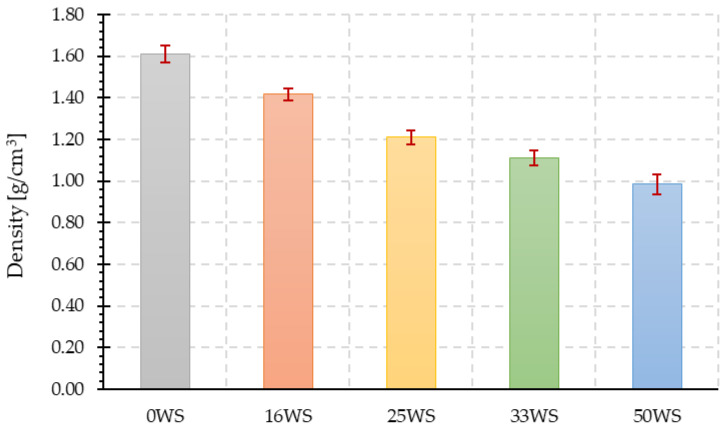
The density of the tested geopolymer composites.

The results of density measurements for all tested geopolymer compositions are shown in Figure 7. Ground walnut shells cause a decrease in the density of the fly ash-based geopolymer. The higher the content of ground walnut shells in the composites, the lower the density of the composites. For the composite made with fly ash and ground walnut shells in a 1:1 ratio (50WS), the density decreased by about 49% compared with the reference sample (0WS).

Sarmin et al. [29] showed in their work that for fly ash and metakaolin-based geopolymer composites, the addition of different types of wood aggregates (wood particles, wood flour, and wood fibers) also resulted in lower densities compared with samples without wood aggregates, whose densities oscillated around 1.5 g/cm^3^. However, for geopolymer composites with wood flour, wood fibers, and wood particles, the average density values were about 1.4 g/cm^3^, 1.3 g/cm^3^, and 1.2 g/cm^3^, respectively. Similar results were obtained in works by Chen et al. [60], Alomayri et al. [30], and Hakamy et al. [35], who showed that there is a decrease in the density of geopolymer composites with the increase in sweet sorghum content or fiber content.

**Figure 8:**
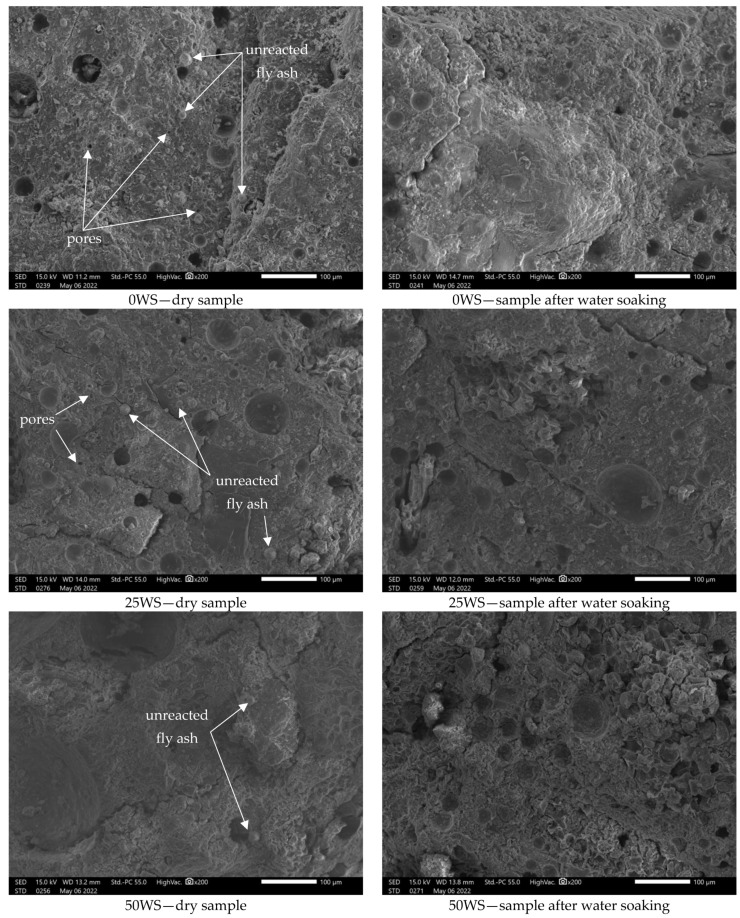
Example SEM images of the structure of a reference sample (0SW) and two tested geopolymer compositions with different contents of ground walnut shells (samples 25SW and 50SW); microphotographs of dry samples (not treated with water) and samples after soaking tests.

Figure 8 shows exemplary scanning microscope (SEM) micrographs of the structure of a reference sample (0WS) and two tested geopolymer composites with different content of ground walnut shells (samples 25WS and 50WS). The microstructure of dry samples (not treated with water) and samples after soaking tests were analyzed.

In the microphotographs presented above, one can observe typical features of fly ash-based geopolymers, described in the literature [61], small, dissolved fly ash particles coexisting with unreacted spheroidal fly ash particles, and sand grain particles, which are distributed in the geopolymer gel. The morphology of the composites studied was strongly influenced by the proportion of ground walnut shells, with an increase in which there was a decrease in both pore size and their proportion in the structure, while the presence of larger voids after trapped air could be observed. Moreover, the higher the content of ground walnut shells in geopolymers, the smaller the amount of unreacted fly ash in the structure. For all the tested geopolymer compositions, a significantly lower amount of unreacted spheroidal fly ash particles in the geopolymer matrix can be observed after saturation tests, which is most likely related to their leaching by water during the test.

Ye et al. [62] observed similar microstructures for geopolymer composites with the addition of wood flour, with samples with high wood flour content found to have a large amount of unreacted fly ash particles partially attached to the wood flour surface.

As demonstrated in their work by Sarmin et al. [29], also in the case of geopolymer composites based on fly ash and metakaolin, in which wood flour was used as an additive, a lower proportion of porosity was observed in their structure in comparison with the geopolymer without any added wood components. On the other hand, geopolymers with wood fiber additives were characterized by the highest porosity.

### 3.4. Compressive and Flexural Strength

**Figure 9:**
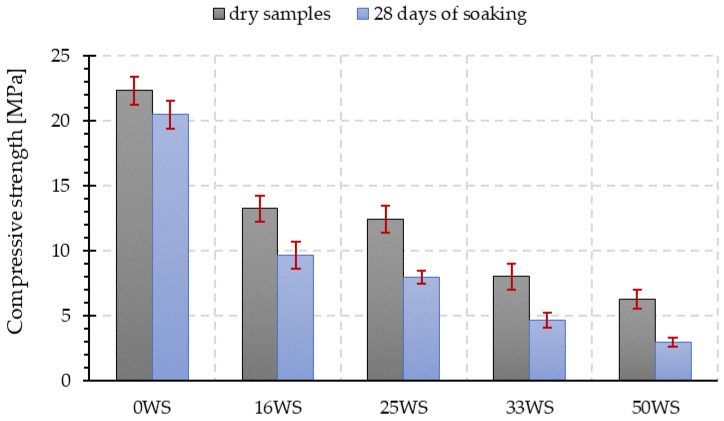
Compressive strength of tested geopolymer composites not exposed to water (dry specimens) and after 28 days of water absorption testing.

Figure 9 shows the compressive strength test results of the tested geopolymer composites not exposed to water (dry) and after the water absorption test. The introduction of ground walnut shells in place of sand contributes to a significant decrease in the mechanical properties of the tested geopolymers. The lower the obtained values of compressive strength were, the higher content of ground walnut shells in the composite was. As a result, for the composite in which the ratio of fly ash to ground walnut shells is 1:1 (50WS), the obtained value of compressive strength is more than three times lower compared with the compressive strength of the sample made of fly ash and sand in a ratio of 1:1, without walnut addition (0WS).

Regardless of the composition, the compressive strength values for all the composites tested decreased after water absorption testing for 28 days. The higher the proportion of ground walnut shells in the geopolymer composite, the higher the decrease in these values was. The highest decrease of about 71% was observed for the sample with 50% by weight of ground walnut shells (50WS). The percentage decrease in the compressive strength for the remaining 16WS, 25WS, and 33WS composites compared with the 0WS sample was about 40%, 44%, and 64%, respectively.

**Figure 10:**
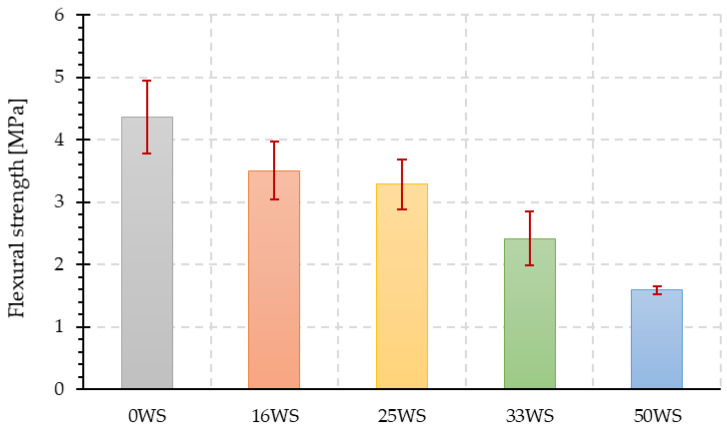
Flexural strength of tested geopolymer composites.

Figure 10 shows the results obtained from the flexural strength measurements for all tested geopolymer compositions. Similarly, as in the case of compressive strength, with the increase in the proportion of ground walnut shells, there is a significant decrease in flexural strength of the tested samples. For the composite, in which the ratio of the proportion of fly ash to ground walnut shells was 1:1 (50WS), the obtained value of flexural strength is more than two times lower (about 63%) compared with the flexural strength of the reference sample (0WS). The percentage decrement of 16WS, 25WS, and 33WS compared with the 0WS mixture was about 19%, 24%, and 44%, respectively.

The decrease in the strength properties of the studied geopolymer composites may be caused by a reduction in the fluidity of the mortar caused by the addition of ground walnut shells, which in turn causes an increase in the ratio of empty spaces in their structure. Moreover, as shown in the example of the glass fibers addition in fly ash-based geopolymer composites [63], the reduction in strength properties could have been caused by the reaction of the additives (in the present work of the ground walnut shells) with the matrix.

As presented by Sarmin et al. [29] also, the addition of various types of wood aggregates, i.e., wood particles, wood flour, and wood fibers, adversely affect the compressive strength of geopolymer composites based on fly ash and metakaolin; the samples with the addition of wood aggregates showed compressive strength in the range of 17.15 MPa to 38.40 MPa, while the reference samples achieved compressive strengths ranging from 35.73 MPa to 76.49 MPa. Similarly, Chen et al. [60] indicated a decrease in compressive strength of fly ash-based geopolymer composites because of the introduction of sweet sorghum additive. Ye et al. [62] showed that for high wood flour content (10 wt.%, 15 wt.%, and 20 wt.%), there is also a significant reduction in both compressive strength and flexural strength of fly ash-based geopolymer composites. Also, Ribeiro et al. [64] have shown that the addition of bamboo fibers causes a decrease in the mechanical properties of geopolymers.

### 3.5. Thermal Conductivity

**Figure 11:**
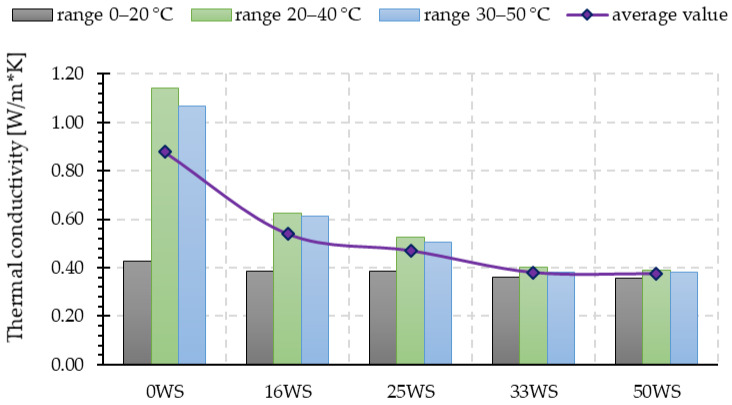
Thermal conductivity over the ranges: 0–20 °C, 20–40 °C, and 30–50 °C for the tested geopolymer composites.

Figure 11 shows the measured thermal conductivity values in three ranges (0–20 °C, 20–40 °C, and 30–50 °C) for the tested geopolymers. Replacement of sand with ground walnut shells significantly contributes to the reduction in thermal conductivity of the tested fly ash-based geopolymers. The lowest value of the average thermal conductivity was characteristic for the samples that had the highest proportion of ground walnut shells in their composition (33WS and 50WS), for which the value obtained was more than 50% lower compared with the average thermal conductivity obtained for the reference sample (0WS).

As indicated in the literature [65], generally, the thermal conductivity of conventional cement is higher compared with geopolymers. For example, metakaolin-based geopolymers, having a density of 1.43–1.89 g/cm^3,^ have a thermal conductivity of 0.55–0.65 W/m*K, while at a geopolymer density of 0.27 g/cm^3^, the thermal conductivity 0.067 W/m*K can be achieved, but can increase to a value of 0.16 W/m*K at a density of 0.35–0.4 g/cm^3^ [66]. Rashad, in his work [67], reported a study in which the thermal conductivity for geopolymer mortar was 0.93 W/m*K.

## 4. Conclusions

In this paper, the effect of the addition of ground walnut shells as a substitute for sand on selected properties of fly ash-based geopolymer composites was investigated. Based on the analysis of the obtained test results, the following conclusions can be drawn:

- The waste in the form of ground walnut shells with success can be used as a substitute for quartz sand in the production of geopolymer mortars;
- The use of ground walnut shell additive contributes to the increase in absorption properties of fly ash-based geopolymers; with the absorption being higher, the higher the proportion of the additive in the composite;
- The addition of ground walnut shells into geopolymers allows for a very large reduction in efflorescence on their surface, which should be considered a positive effect because, as is known, the appearance of efflorescence in the case of geopolymers can negatively affect their mechanical properties;
- The occurrence of efflorescence may have been caused mainly by the use of low reactive fly ash. Part of the Na present in the alkaline solution (even a small part) was not completely combined with the fly ash and was, therefore, free to react with the CO_2_ present in the atmosphere;
- The density of geopolymer composites based on fly ash is significantly reduced by the introduction of ground walnut shells in place of sand, allowing for a reduction in its value by almost half with the complete replacement of sand with ground walnut shells;
- Compressive strength and flexural strength are reduced by using ground walnut shells. The reduction in mechanical properties was greater with the higher weight proportion in the mixture used to make the geopolymer composites.
- Partial or complete replacement of sand by ground walnut shells in geopolymer composites based on fly ash allows for a significant reduction in their thermal conductivity (over 50% compared to the reference sample), which makes it possible to use these composites as insulation materials.

## Figures and Tables

**Table 1:** Determination of samples along with weight/volume share of solid raw materials.

Sample ID	The Proportion of Solid Components (% by Weight/by Volume)		
	Fly Ash	Sand	Ground Walnut Shells
0WS	50/36.6	50/63.4	-
16WS	50/43.7	33.33/37.9	16.67/18.5
25WS	50/46.7	25/27.0	25/26.4
33WS	50/41.0	16.67/47.4	33.33/11.6
50WS	50/54.1	-	50/45.9

## Data Availability

Not applicable.
